# Differences in hindlimb morphology of ducks and chickens: effects of domestication and selection

**DOI:** 10.1186/s12711-015-0166-9

**Published:** 2015-11-17

**Authors:** Brendan M. Duggan, Paul M. Hocking, Tobias Schwarz, Dylan N. Clements

**Affiliations:** The Roslin Institute and Royal (Dick) School of Veterinary Studies, University of Edinburgh, Easter Bush, Midlothian, EH25 9RG UK

## Abstract

**Background:**

Poultry account for the most numerous species farmed for meat and have been subject to intense selection over approximately 60 generations. To assess morphological changes which have occurred in the avian leg due to selection for rapid growth and high meat yields, divergent lines of chicken (*Gallus gallus*) and duck (*Anas platyrhynchos*) were studied between 3 and 7 weeks of age. For each line, femoral and tibiotarsal morphology was recorded using computed tomography scanning and tibiotarsal bone quality measures (stiffness, bending stress and porosity) were assessed.

**Results:**

In chicken and duck, divergence in hindlimb morphology has occurred in the commercial meat lines compared to their lighter conspecifics. As expected, the differences were largest between species. Leg development nears completion much earlier in ducks than in chickens. Duck tibiotarsi showed a large degree of lateral curvature, which is expected to affect foot position during swimming and walking, and thus to influence gait. All lines have adapted their tibiotarsal morphology to suit the loading forces they experience; however bone quality was found to be poorer in chickens.

**Conclusions:**

We demonstrate that intensive selection for growth rate in both chickens and ducks has resulted in leg morphology changes, which are likely to influence gait. Ducks represent an interesting compromise of adaptation for efficient locomotion in two media—on land and in water. Some aspects of bone morphology in the duck, such as lateral curvature of the tibiotarsus, may result from adaptation to swimming, which potentially imposes limitations on terrestrial locomotion.

**Electronic supplementary material:**

The online version of this article (doi:10.1186/s12711-015-0166-9) contains supplementary material, which is available to authorized users.

## Background

Poultry are the most numerous animals farmed for meat. The Food and Agriculture Organisation of the United Nations reported that over 60 billion chickens and 2 billion ducks are produced worldwide each year, with these figures increasing annually [1]. During approximately 60 generations of selection, the meat type (broiler) bird has undergone intensive selection for rapid growth and increased pectoral muscle mass [2, 3]. For example, the broiler chicken has experienced a 300 % increase in body mass over this period [4]. One unwanted side effect of this genetic gain has been an increased incidence of locomotion problems (termed “leg weakness”) [5].

Various studies have reported figures for the prevalence of leg weakness in broiler chickens that range from 15 to 30 % [4, 6–8]. Accurate figures are difficult to obtain due to variation between studies in the genotypes and gait scoring systems used, the age at which birds are assessed and management factors [9]. While recent reliable information on the prevalence of leg weakness in poultry is not available, it is likely that this issue causes economic losses to the producer [10]. Leg weakness in livestock is also a welfare issue; since it has been associated with pain [11–13] and modified behaviour [14, 15]. However, selection strategies over the last 25 years have addressed some leg weakness issues [16]. Data on the prevalence of welfare issues in domestic ducks are scarce [17, 18] but given that, in duck and chicken breeding, selection intensities and achieved growth rates and carcass weights are similar, it is likely that locomotion problems also exist to some extent in the domestic duck.

The causes of poor gait are varied. In broiler chickens, an increase in pectoral muscle mass has shifted the body’s centre of mass cranially which is associated with relatively poor stability [19, 20]. Bone deformities may also play a role: valgus, varus and torsional deformities are generally seen in the tibiotarsus and have previously been associated with gait abnormalities [21]. Some gait problems can be due to bone fractures, which either occur due to trauma or are secondary to other bone pathologies [21]. Fracture risk is often linked to bone quality; cortical bone has been shown to be less well mineralised and more porous in broiler chickens that are selected for rapid growth than in slower growing lines [22, 23]. In Pekin ducks, tibiotarsal bone mineral density seems to have remained within a similar range during the last two decades, although tibiotarsal length and body mass have increased [24].

The aim of this study was to assess skeletal changes which have occurred in the Pekin duck during its selection for rapid growth and to compare these with different lines of chickens. Since such a vast number of fast-growing ducks and chickens are reared for meat each year, a better understanding of these birds’ gait may lead to welfare improvements on a large scale. The duck leg represents an interesting compromise of adaptation for efficient locomotion in two media, i.e. on land and in water; it is expected that adaptations which are beneficial to swimming will create a leg morphology which differs from that of a strictly cursorial species such as the chicken. To represent divergent lines of chicken, broiler chickens were used as an example of a line selected for rapid growth, and layers were used to represent a growth rate more similar to their ancestral phenotype, the red jungle fowl (*Gallus gallus*). For Pekin ducks, a commercial hybrid and two breeding lines were used as examples of high growth rate birds; these were compared to their ancestral phenotype, the mallard (*Anas platyrhynchos*). Selection for high feed efficiency and breast muscle yield in both species was anticipated to have led the heavier meat lines to diverge from their lighter conspecifics for skeletal morphology. Both the Pekin duck and the broiler chicken have undergone intense selection for breast muscle mass over many decades. This selection has led to a cranial shift in the body’s centre of mass, thus altering the loading forces which act on the legs of both lines [2]. As well as recording data on leg morphology, allometric scaling patterns of various traits were also compared; normally, aspects of hindlimb morphology would be expected to scale isometrically (that is, with geometric similarity) to body mass. However, due to differences in the natural habitats and locomotor modes of the ancestors of chickens and ducks, some deviations from isometry were expected.

## Methods

### Animals and husbandry

A total of 216 birds of different lines were culled at three ages in two separate experiments. During the first experiment, 36 broiler chickens (Ross 308), 36 layer chickens (Lohman Brown) and 36 Pekin ducks (Cherry Valley commercial hybrid) were raised in walled research pens. The second experiment used the same pens to house 36 Pekin ducks of a male line, 36 Pekin ducks of a female line (both Cherry Valley breeding stock) and 36 mallards (Hy-Fly Game Hatcheries, Poulton-le-Fylde, UK). The male Pekin line, which are the eventual male grandparents of the commercial hybrid line, are selected primarily for growth and feed efficiency while the female Pekin line, which are the female grandparents of the commercial hybrid line, were selected for fertility, as well. Both breeding lines contained equal numbers of both males and females.

Birds were raised following industry guidelines as much as possible. All birds were initially housed from day of hatch under brooder lamps in a single pen per line to regulate temperature. At 7 days, birds were randomly allocated in a randomised block design to two blocks of nine pens, separated by a 3 m passage. Each pen (2.16 m^2^) contained four males and four females housed in an area of 0.27 m^2^ per bird, increasing to 0.36 m^2^ per bird from 21 days and 0.54 m^2^ per bird from 35 days onward as birds were removed for measurement. The lighting regime was 23 h light:1 h dark at hatch, reducing by 1 h light per day for the first 7 days and remaining at 16 h light:8 h dark thereafter. The mean light intensity in each pen was 120 lux. Barn temperature was 25 °C at 2 weeks, decreased to 24 °C at 3 weeks, 22 °C at 4 weeks and remained at 20 °C from 5 weeks until termination, as per industry guidelines. Experiment 1 used wood shavings as substrate since this is the industry norm for chickens. Experiment 2 used straw as substrate, as is the case on most UK duck farms. All birds were provided with food and water ad libitum. Broilers were given a commercial starter feed for the first 10 days, grower feed from days 11 to 35 and finisher feed from day 36 onwards. Layers were fed on a commercial starter feed for the first 35 days before transferring to grower feed from day 36 onwards. All duck lines in both experiments were fed on a starter feed until day 10 and then a grower feed thereafter; both duck diets were supplied by the breeding company.

The use of animals for this study was approved by the University of Edinburgh Ethics Committee.

### Data collection

At three ages (21, 35 and 49 days), two randomly selected birds (one male and one female) from each pen (six males and six females per line) were euthanatized by intravenous sodium pentobarbital injection (Euthatal, Merial, Toulouse, France) and immediately dissected. These ages were chosen to cover the typical lifespan of a production bird of both species. Body mass of each bird was recorded 2 days prior to euthanasia. The left pectoral muscle and thigh and shank muscle groups of the femur and tibiotarsus were dissected out and weighed. Both tibiotarsi and femora were left intact at the stifle joint and stored at −20 °C for future measurement. At a later date, the bones were thawed and the left tibiotarsus and femur were evaluated with a computed tomography scan (CT). A helical 4-slice CT unit (Somatom Volume Zoom, Siemens, Germany) was used. For each scan, six legs were laid parallel to each other in supine position (cranial aspect facing upward), and scanned along their full length using a 1 mm slice width. The tibiotarsus was parallel to the table while the femur, still attached to the tibiotarsus, was at an approximately 10° angle (with the proximal end of the femur closer to the table than the distal end). Bone morphology was assessed using a 3D multi-planar reconstruction in dedicated DICOM viewing software (OsiriX, Geneva, Switzerland, version 5.8.5—32 bit). Morphological measurements for both femur and tibiotarsus include functional length, diameter and cortical cross-sectional area at the mid-diaphysis, curvature in both frontal and sagittal planes and torsion. Detailed methods are in Additional file 1.

Bone breaking tests were performed using an LRX Materials system running ‘Nexygen 2.2’ software (Lloyds Instruments, Bognor Regis, UK) to assess stiffness and ultimate breaking strength. Stiffness is a measure of the force required to displace the mid-diaphysis of a bone by a known distance when the ends are fixed. Ultimate breaking strength is the maximum load the bone can withstand before breaking. Compress to rupture tests were carried out on the right tibiotarsus using a three-point-bending jig, i.e. each bone was balanced in supine position on two curved rests 10 mm in diameter and 30 mm apart with a downward force (also curved, 10 mm diameter) centrally applied at the mid-diaphysis at a rate of 30 mm/min until rupture.

A 1.5 cm portion of the mid-diaphysis was cut from the broken (right) tibiotarsus using a circular bone saw and sent for mineral content analysis (DM Scientific, Thirsk, UK) to determine bone mineral density. A 1 cm section was also cut from the mid-diaphysis of the left tibiotarsus for porosity assessment by histology (see Additional file 1).

### Data analysis

Bending stress (*B*) is a measure of the maximum force experienced by the tibiotarsi before breaking, corrected for the anatomical shape of the bone. Bending stress was calculated using the formula:

$$B = \frac{My}{I},$$

where *M* is the bending moment (the maximum load applied to the bone multiplied by the distance over which it is applied), *y* is the distance from the cross-sectional centre of mass in the direction of loading (in this case, the outer semi-minor axis as the cross-section is a hollow ellipse) and *I* is the second area moment of inertia.

Full details of the calculation are in Additional file 1.

A split-plot statistical model was analysed using Genstat statistical software (version 16.1.0.10916 (64-bit), VSN International, Ltd.) using ANOVA, with effects for pen nested within block and treatment effects of genetic line, age and sex. Because some conditions differed between experiments, the six lines were not analysed together; separate ANOVA were performed to compare the lines from Experiment 1 (broiler chicken, layer chicken and Pekin commercial hybrid) and the lines from Experiment 2 (male Pekin line, female Pekin line and mallard).

Scaling relationships through ontogeny were analysed by regressing the log of each trait against the log of body mass. Since both body mass and bone/muscle measurements were expected to contain some error, reduced major axis (also called Model II) regression was performed. The slope (scaling exponent) of the resulting regression equation for each trait was compared to the expected scaling exponent for that trait. Assuming that traits scale isometrically (that is, they grow with geometric similarity to body mass) and considering that the predictive trait (body mass) is volumetric, lengths were expected to scale to body mass^0.33^, measurements of area were expected to body mass^0.67^ and mass measurements were expected to scale to body mass^1^. Non-dimensional measurements (such as bone torsion angles) were expected to scale to body mass^0^; in other words, they were not expected to change as body mass increased. See Allen et al. [25] for a detailed description of this analysis.

## Results

Least squares means and standard errors of the differences for each trait in all lines at all ages are in Additional file 2.

**Fig. 1:**
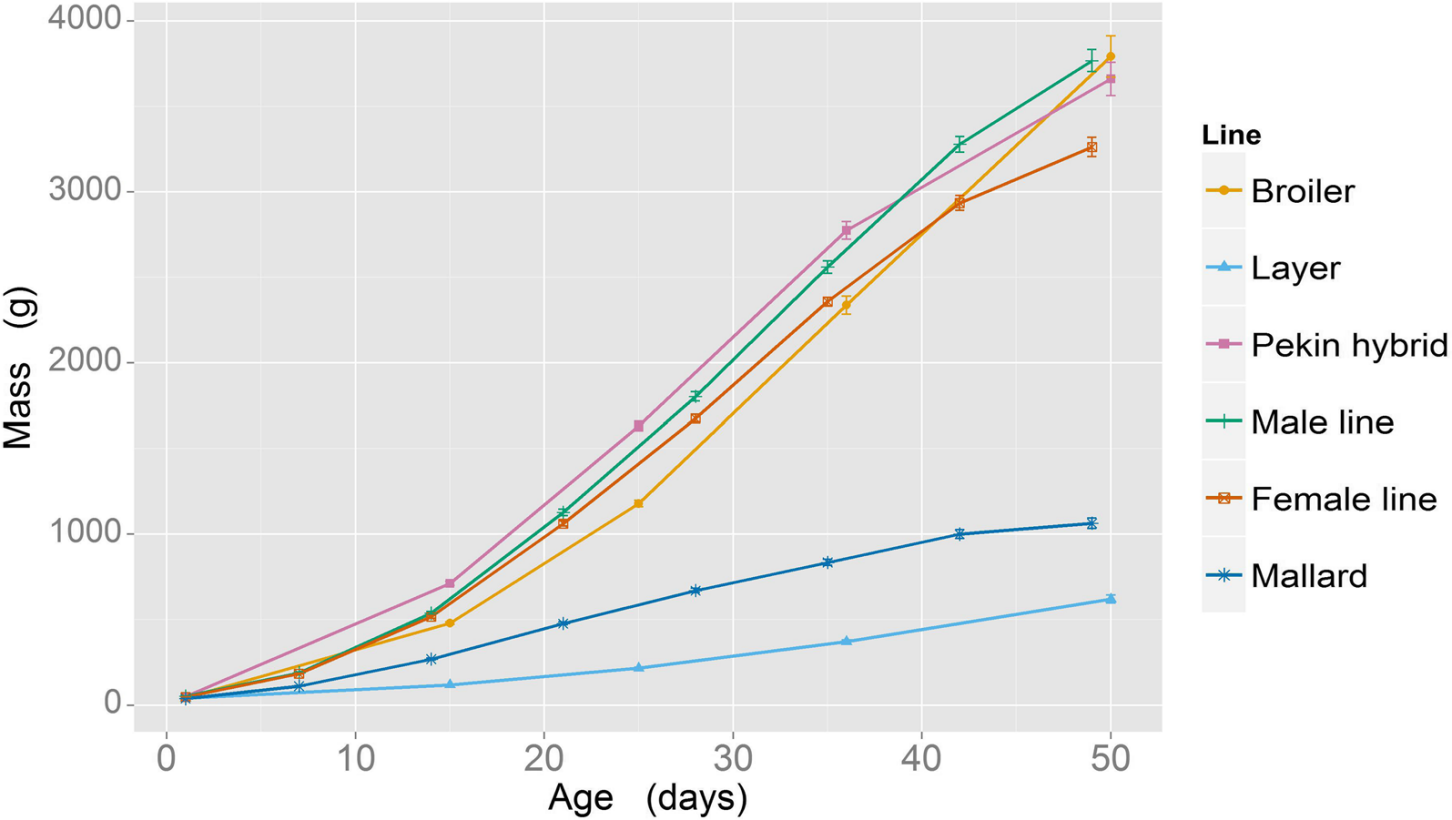
Body mass measurements (means and standard errors) from hatch to 7 weeks of age. Data are combined from Experiment 1 (broiler chicken, layer chicken, Pekin duck commercial hybrid) and Experiment 2 [male Pekin line (M. line), female Pekin line (F. line) and mallard]

Figure 1 shows changes in body mass for each line over 7 weeks. There was a significant difference in body mass between lines in both experiments (*P* < 0.001). The broiler chicken and all three Pekin duck lines grew at a faster rate than both the layer chicken and the mallard (*P* < 0.001).

**Fig. 2:**
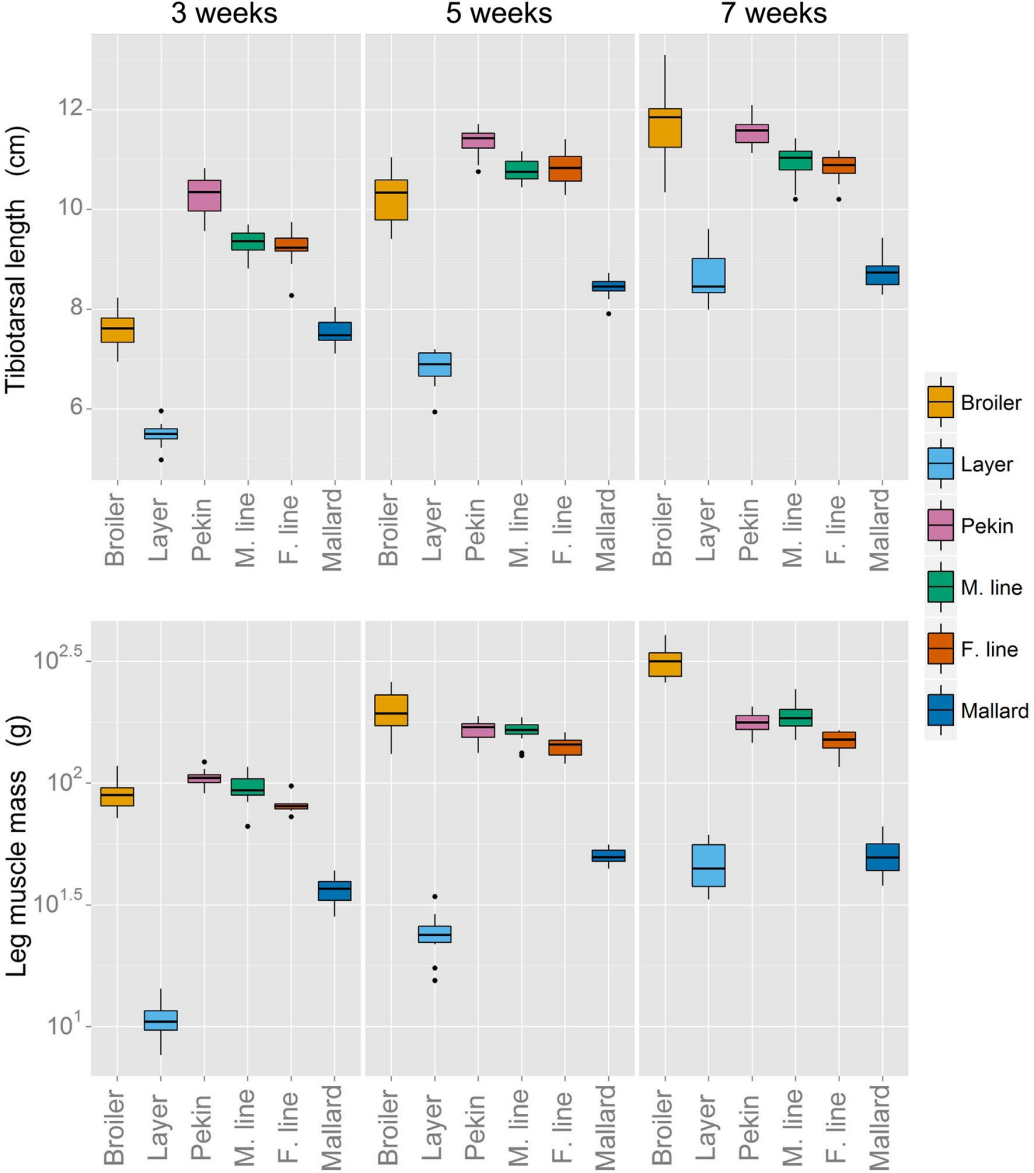
Tibiotarsal length and leg muscle mass from 3 to 7 weeks of age. Leg muscle mass (drumstick and thigh muscles) is presented using a log-scale for clarity. The *upper* and *lower boxplot whiskers* extend to within 1.5 times above and below the interquartile range, respectively. *Dots* outside this range are marked as *outliers*. Note the lack of growth in both bone length and muscle mass in all duck lines after 5 weeks of age

Differences in leg bone length mirrored those of body mass; the tibiotarsus (Fig. 2a) was significantly shorter in the layer chicken and mallard compared to the broiler chicken and Pekin lines (*P* < 0.001). There was an age effect (*P* < 0.001) i.e. duck lines in each experiment showed a decline in tibiotarsal growth from 5 weeks of age whereas chicken tibiotarsi continued to grow throughout the experiment. When analysed allometrically, chicken tibiotarsi grew with positive allometry and the tibiotarsi of all four duck lines grew with negative allometry (Table 1). A sex effect (*P* < 0.001 in Experiment 1 and *P* = 0.002 in Experiment 2) was also observed; males of all lines had longer tibiotarsi than females. In Experiment 1 an age by sex interaction was observed (*P* < 0.001) i.e. females (broiler chicken and Pekin hybrid, but not layer chicken) had longer tibiotarsi than males at 3 weeks but not at 5 and 7 weeks of age (*P* < 0.001).

**Table 1:** Allometric analysis of reduced major axis regressions

	Expected slope	Slope	Lower CI	Upper CI	R^2^
*Tibiotarsal length*					
Broiler	0.33	0.40 (+)	0.37	0.44	0.93
Layer	0.33	0.43 (+)	0.41	0.46	0.98
Pekin hybrid	0.33	0.17 (−)	0.15	0.20	0.78
Pekin male line	0.33	0.15 (−)	0.13	0.18	0.84
Pekin female line	0.33	0.17 (−)	0.15	0.20	0.82
Mallard	0.33	0.21 (−)	0.18	0.23	0.88
*Femoral length*					
Broiler	0.33	0.33 (=)	0.30	0.35	0.95
Layer	0.33	0.41 (+)	0.38	0.44	0.97
Pekin hybrid	0.33	0.23 (−)	0.20	0.26	0.85
Pekin male line	0.33	0.22 (−)	0.20	0.24	0.93
Pekin female line	0.33	0.21 (−)	0.18	0.23	0.88
Mallard	0.33	0.24 (−)	0.21	0.26	0.91
*Leg muscle mass*					
Broiler	1	1.15 (+)	1.09	1.21	0.98
Layer	1	1.41 (+)	1.34	1.48	0.98
Pekin hybrid	1	0.70 (−)	0.62	0.80	0.86
Pekin male line	1	0.64 (−)	0.58	0.71	0.91
Pekin female line	1	0.59 (−)	0.53	0.66	0.90
Mallard	1	0.59 (−)	0.48	0.72	0.68
*Tibiotarsal torsion*					
Broiler	0	−0.08	−0.11	−0.06	0.10*
Layer	0	0.05	0.03	0.07	0.00*
Pekin hybrid	0	−0.21	−0.28	−0.15	0.22
Pekin male line	0	−0.13	−0.17	−0.10	0.35
Pekin female line	0	−0.12	−0.15	−0.09	0.44
Mallard	0	−0.19	−0.25	−0.15	0.43
*Femoral torsion*					
Broiler	0	0.09	0.07	0.13	0.01*
Layer	0	−0.07	−0.10	−0.05	0.02*
Pekin hybrid	0	−0.17	−0.22	−0.13	0.52
Pekin male line	0	−0.11	−0.14	−0.09	0.59
Pekin female line	0	−0.10	−0.13	−0.08	0.47
Mallard	0	−0.11	−0.15	−0.08	0.24
*Tibiotarsal cortical area*					
Broiler	0.67	0.81 (+)	0.68	0.96	0.75
Layer	0.67	0.77 (+)	0.67	0.88	0.86
Pekin hybrid	0.67	0.60 (=)	0.49	0.74	0.66
Pekin male line	0.67	0.34 (−)	0.29	0.41	0.74
Pekin female line	0.67	0.31 (−)	0.26	0.37	0.86
Mallard	0.67	0.48 (−)	0.41	0.55	0.80
*Femoral cortical area*					
Broiler	0.67	0.68 (=)	0.57	0.81	0.75
Layer	0.67	0.80 (+)	0.71	0.90	0.89
Pekin hybrid	0.67	0.47 (−)	0.35	0.64	0.24
Pekin male line	0.67	0.19 (−)	0.14	0.27	0.02*
Pekin female line	0.67	0.21 (−)	0.15	0.29	0.07*
Mallard	0.67	0.35 (−)	0.28	0.45	0.53
*Tibiotarsal stiffness*					
Broiler	0.67	0.63 (=)	0.54	0.73	0.81
Layer	0.67	1.28 (+)	1.14	1.43	0.90
Pekin hybrid	0.67	0.79 (=)	0.66	0.95	0.73
Pekin male line	0.67	0.78 (=)	0.67	0.90	0.81
Pekin female line	0.67	0.77 (=)	0.66	0.90	0.81
Mallard	0.67	0.79 (=)	0.62	1.00	0.52
*Tibiotarsal maximum load*					
Broiler	0.67	0.78 (+)	0.68	0.89	0.85
Layer	0.67	1.13 (+)	0.99	1.27	0.88
Pekin hybrid	0.67	1.01 (+)	0.89	1.15	0.86
Pekin male line	0.67	0.67 (=)	0.59	0.76	0.87
Pekin female line	0.67	0.74 (=)	0.65	0.84	0.88
Mallard	0.67	0.88 (+)	0.79	0.99	0.89

Slopes and R^2^ values for various bone traits are provided, along with their 95 % confidence intervals. Regressions presented here were significant (*P* < 0.01) with the exception of those marked *. All length measurements that are regressed against body mass have an expected slope of 0.33 and measurements of areas have an expected slope of 0.67. Angular measurements such as torsion are expected to have a slope of 0. The symbols next to each slope indicate positive allometry (+), negative allometry (−) or isometry (=)

**Fig. 3:**
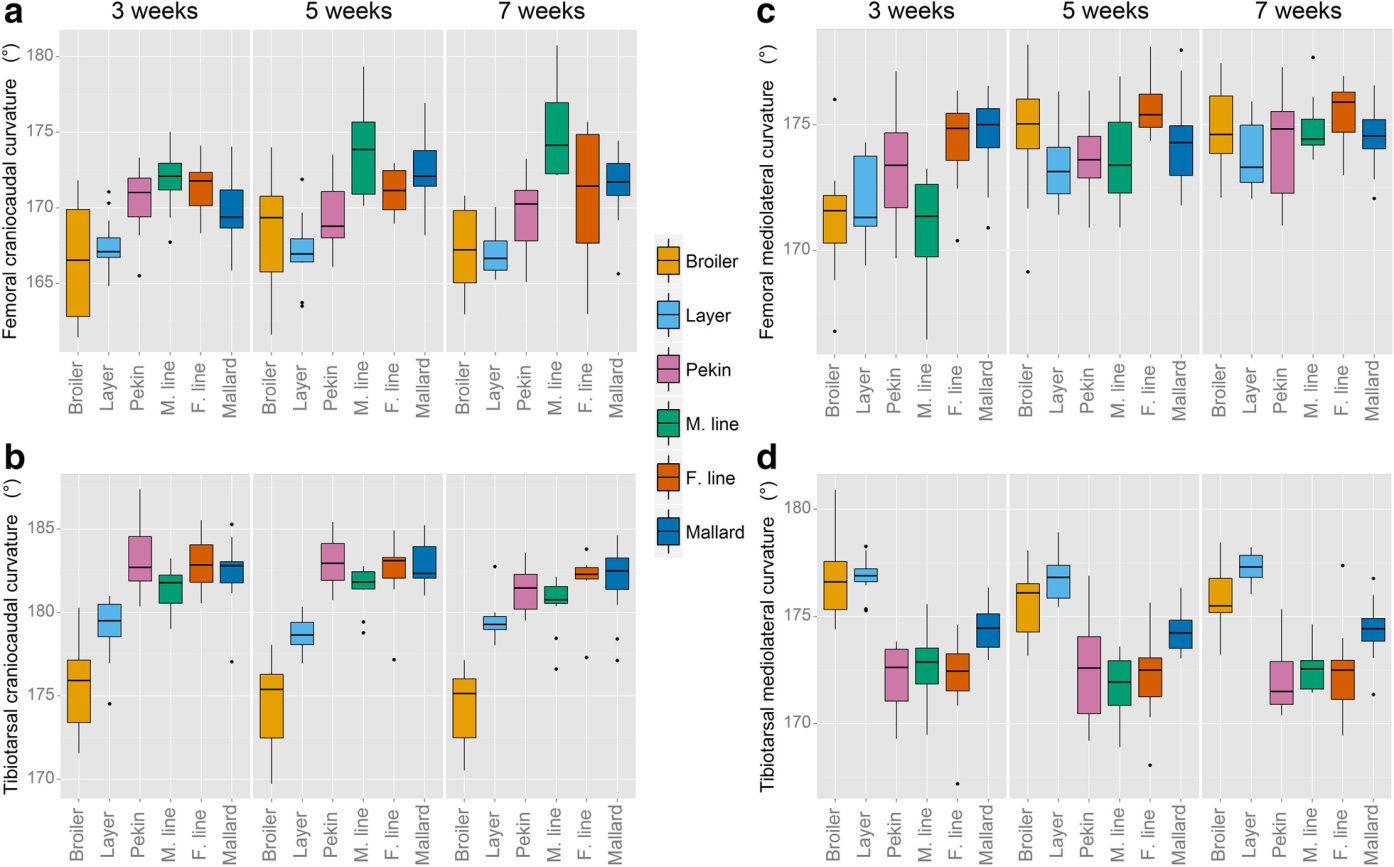
Cranio-caudal and medio-lateral curvature of the femur and tibiotarsus (in degrees). 180° represents a straight bone. A value below 180° represents cranial and lateral bending in the craniocaudal and mediolateral planes, respectively

In the cranio-caudal plane, the tibiotarsus of the broiler chicken was significantly more curved (cranially) than the tibiotarsus of the layer chicken, which was in turn more cranially curved than that of the Pekin hybrid (*P* < 0.001). In Experiment 2, the mallard tibiotarsus displayed significantly more caudal curvature than that of the male Pekin line (*P* = 0.013) but did not differ from that of the female line (Fig. 3b). Male birds in Experiment 1 exhibited greater cranio-caudal curvature of their tibiotarsi than females. Both species differed in the direction of tibiotarsal curvature in this plane, i.e. all four duck lines curved caudally whereas both chicken lines curved cranially. In the medio-lateral plane (Fig. 3d), the tibiotarsi of both the broiler chicken and Pekin duck displayed greater lateral curvature than their lighter conspecifics; however, this difference was statistically significant (*P* < 0.001) only between the mallard and Pekin breeding lines. In this plane, the duck tibiotarsi were more laterally curved than those of both chicken lines (*P* < 0.001).

**Fig. 4:**
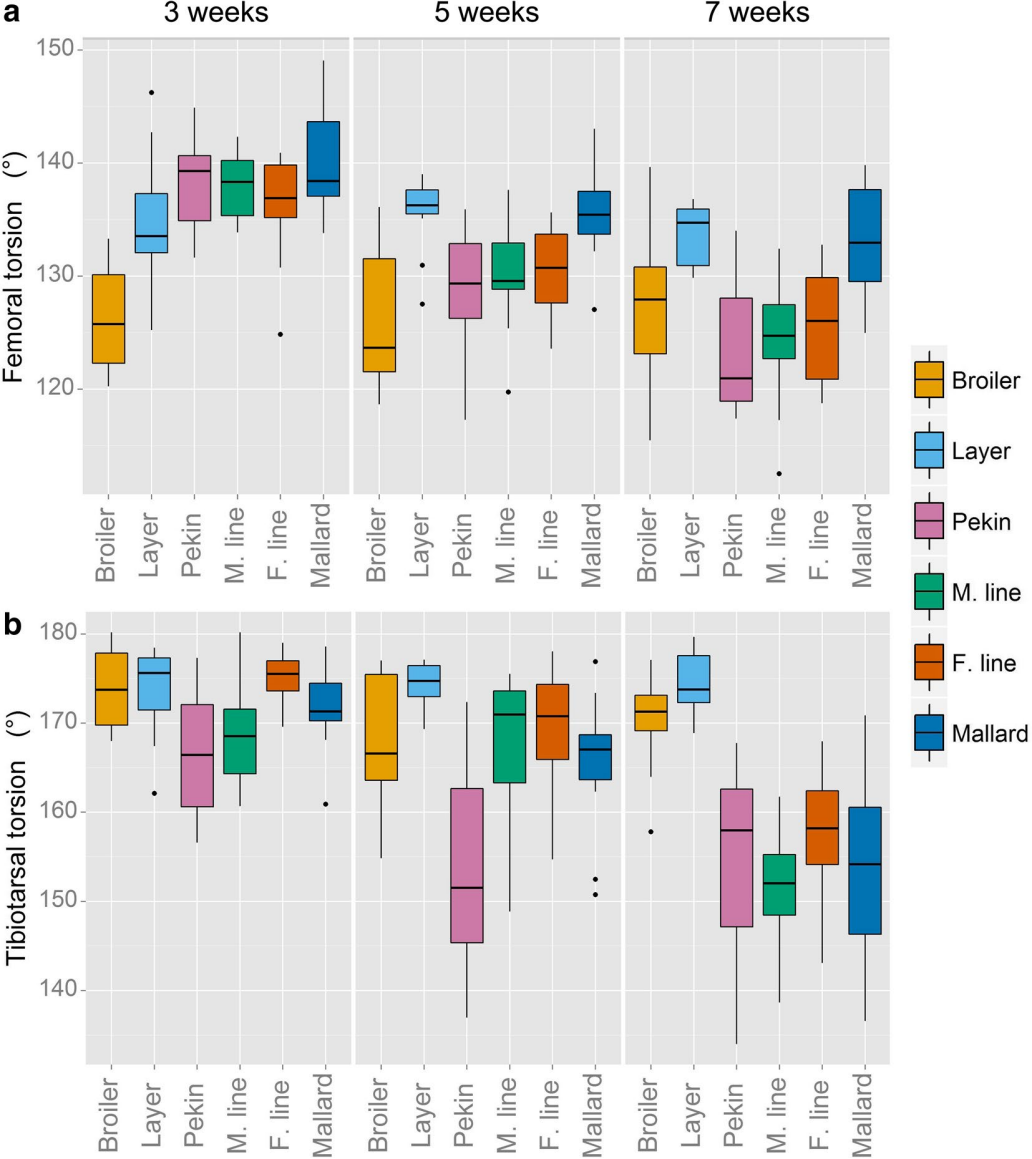
Rotation of the distal part of the femur and tibiotarsus in relation to the proximal part of the femur and tibiotarsus, respectively. Angles at the starting point at 3 weeks of age are based on the relative position of bone landmarks and are not a measure of initial rotation. A decrease in angle after 3 weeks represents external femoral rotation and internal tibiotarsal rotation

Tibiotarsal torsion occurred to a similar extent in both chicken lines. The Pekin hybrid differed significantly from the chicken lines (*P* < 0.001). There was a line by age interaction, i.e. at 3 weeks of age, the Pekin and chicken lines displayed a similar range of tibiotarsal torsion but by 7 weeks of age the distal part of the tibiotarsus of the Pekin hybrid had rotated internally in relation to the proximal tibiotarsus (*P* = 0.005) (Fig. 4b). Internal rotation occurs when the cranial aspect of the distal part of the tibiotarsus turns to face medially. No difference in tibiotarsal torsion was observed between the mallard and Pekin breeding lines in Experiment 2; however, the distal tibiotarsi of the male Pekin line rotated internally to a greater extent than that of the female Pekin line (*P* = 0.024). There was an age interaction (*P* < 0.001) with tibiotarsi in all three duck lines of Experiment 2 rotating internally as they aged. A line by age interaction also occurred in Experiment 1, i.e. the distal tibiotarsi of the Pekin commercial hybrid rotated internally as the bird aged (*P* = 0.005) whereas the tibiotarsi of the chicken lines did not. The R^2^ values from regressions of the log of bone torsion on the log of body mass were very low for both chicken lines (Table 1), suggesting no relationship. In the duck lines, tibiotarsal torsion deviated slightly from isometric growth.

### Tibiotarsal bone quality

**Fig. 5:**
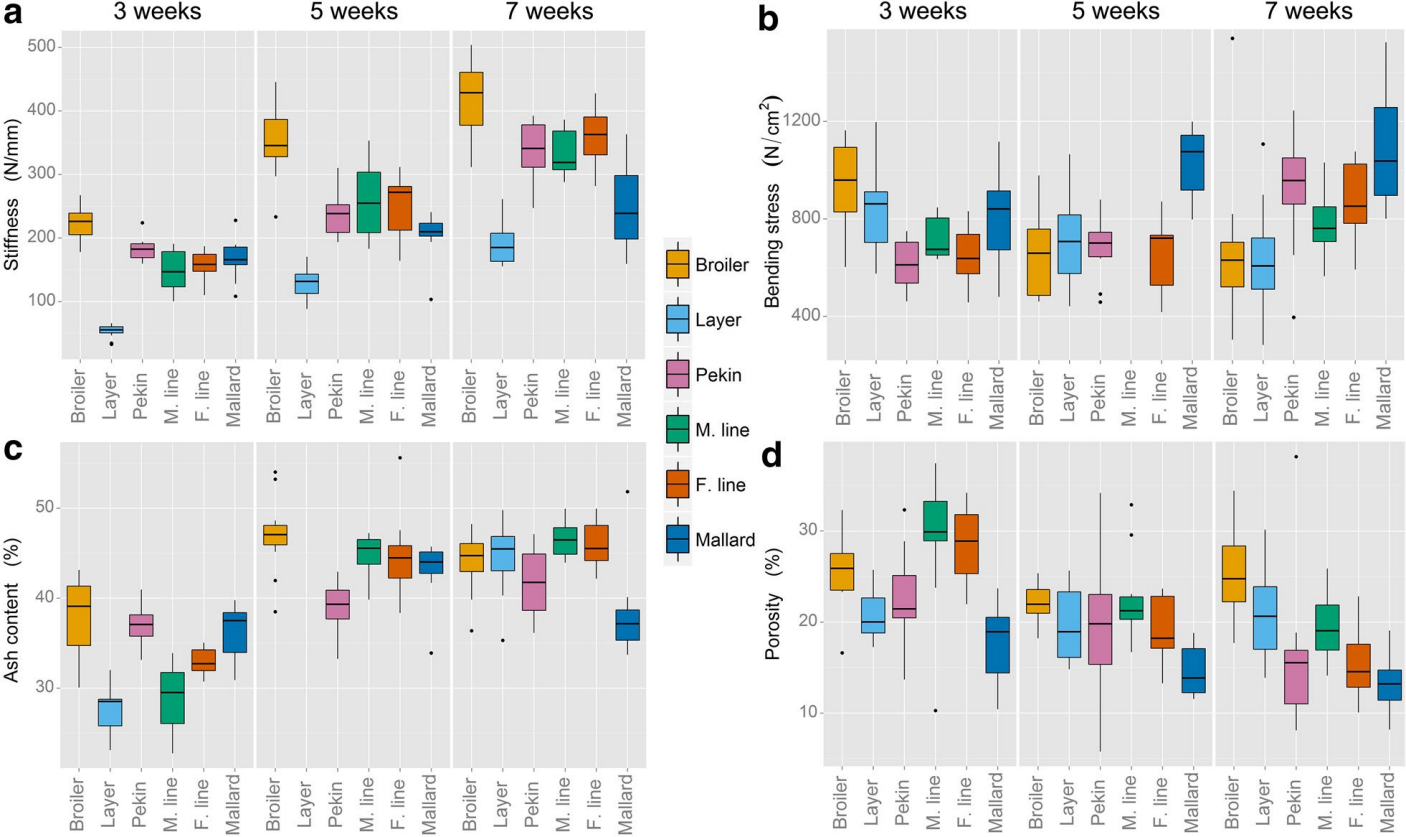
Tibiotarsal bone quality measurements. **a** Stiffness and **b** bending stress were measured on the whole bone. **c** Ash content (wet bone) and **d** porosity were measured on a 1 cm section of the mid-diaphysis. Ash content data for layer chickens at 5 weeks of age and bending stress values for the male Pekin line at 5 weeks of age were excluded due to measurement error

Tibiotarsal stiffness differed significantly within both chickens and ducks (*P* < 0.001). Lines selected for rapid growth had stiffer tibiotarsi than lines with a slow growth (Fig. 5a). There was an age effect in Experiment 2, i.e. the tibiotarsi of the fast-growing Pekin lines had the same stiffness as the mallard at 3 weeks of age but, by 7 weeks, they were significantly stiffer than those of the mallard (*P* < 0.001). Tibiotarsal stiffness scaled isometrically in all lines except for the layer chicken, which displayed very positive allometry (Table 1). Tibiotarsal strength (maximum load to rupture) for all lines at all ages was significantly greater in fast-growing lines compared to their slow-growing conspecifics (*P* < 0.001). Tibiotarsal strength scaled with positive allometry for all lines except for the male and female Pekin breeding lines, which scaled with isometry (Table 1).

Bending stress (Fig. 5b) did not differ significantly between the Pekin hybrid and both chicken lines at all ages. However, there was a line by age interaction (*P* < 0.001), i.e. Pekin hybrid tibiotarsi tolerated greater bending stresses as the animals grew older whereas the bending stresses tolerated by the tibiotarsi of both chicken lines decreased. The mallard tibiotarsi resisted significantly more bending stress than those of the heavier male Pekin line and female Pekin line (*P* < 0.001). There was also an age effect, i.e. the tibiotarsi of all three duck lines tolerated more bending stress as they aged (*P* < 0.001). Data for the male Pekin line at 5 weeks of age was not analysed since these bones moved during loading, causing error.

Data on tibiotarsal ash content for the layer chicken line at 5 weeks of age is not available due to measurement error. The ash content of the bone before drying (Fig. 5c) was significantly greater in the broiler compared with both the layer chicken and Pekin hybrid (*P* < 0.001). All lines had increased bone mineralisation as they aged (*P* < 0.001), although the broiler chicken reached its 7-week level of ash content earlier than the Pekin commercial hybrid (*P* < 0.001). In Experiment 2, all duck lines had increased bone mineralisation as they grew older (*P* < 0.001); however, for the mallard, bone mineralisation increased until 5 weeks of age and then decreased from 7 weeks of age (*P* < 0.001). The molar Ca:P ratio across all lines over all ages ranged from 1.4 to 1.8 (see Additional file 2).

Porosity differed significantly between lines in Experiment 1 (*P* = 0.003), i.e. the tibiotarsi of broiler chicken were more porous at the mid-diaphysis than those of both the layer chicken and Pekin hybrid (Fig. 5d). In Experiment 2, all three duck lines differed significantly in tibiotarsal porosity (*P* < 0.001) with the male line having the highest mid-diaphyseal porosity and the mallard having the lowest. An age interaction was also observed in the duck lines with the tibiotarsi becoming less porous as the birds aged (*P* < 0.001).

### Femoral measurements

The length of the femur was significantly shorter in the layer chicken and the mallard than in the broiler chicken and Pekin male and female lines (*P* < 0.001). Age effects, sex effects and line-by-age interaction effects were observed in both experiments (*P* < 0.001). Femoral length scaled with negative allometry for all duck lines (Table 1). The femoral length of the broiler chicken increased isometrically with body mass and that of the layer chicken showed slightly positive allometric growth.

There was no difference in cranio-caudal curvature of the femur between the broiler and layer chicken in Experiment 1 (Fig. 3a). However, the femora of the chicken lines were more cranially curved than the Pekin hybrid in this plane (*P* < 0.001). In Experiment 2, the femora of the mallard and the female Pekin line displayed more cranial curvature than those of the male Pekin line (*P* = 0.001). In the medio-lateral plane, no significant differences in femoral curvature of the broiler chicken, layer chicken or Pekin duck hybrid were observed. However, there was an age effect (*P* < 0.001), i.e. lateral curvature of the femora of all three lines decreased as the birds aged. In Experiment 2, the femora of the male Pekin line were significantly more laterally curved than those of the female line and the mallard (*P* < 0.001). An age effect and a line-by-age interaction effect were observed in these lines (*P* < 0.001); the femora of the male line became less curved in this plane as the birds aged whereas the female line and mallard maintained the same curvature. By 7 weeks of age, the femora of all lines were curved to a similar degree in the medio-lateral plane (Fig. 3c).

Femoral torsion differed significantly between chicken lines in Experiment 1 (Fig. 4a); the distal femur of the broiler was rotated more externally to the proximal end when compared to that of the layer at all ages (*P* < 0.001). There was also a line-by-age interaction (*P* < 0.001); at 3 weeks of age, the distal femur of the Pekin commercial hybrid was rotated internally compared to that of both chicken lines. However, as the Pekin individuals aged, the distal femur rotated externally, reaching a similar degree of femoral torsion as for the broiler chicken by 7 weeks of age. A similar age interaction occurred in Experiment 2, with the distal femur of all duck lines rotating externally in relation to the proximal femur (*P* < 0.001). The mallard femur underwent less rotation as it aged (*P* = 0.002), reaching a degree or femoral torsion similar to that of the layer chicken by 7 weeks of age.

Femoral cortical area grew isometrically in the broiler chicken and with positive allometry in the layer chicken. The R^2^ value for this trait in duck lines was low, suggesting a weak relationship with body mass.

## Discussion

Both the Pekin duck and broiler chicken have undergone major changes in body size and leg morphology since divergence from their ‘unselected’ conspecifics occurred through artificial selection. Other studies have reported that these changes affect gait [5, 26–28]. Body mass of both chicken and duck meat lines has also considerably increased since divergence from their unselected (or ancestral) phenotype. While the layer chicken cannot be regarded as the broiler’s ancestral phenotype, it has not been submitted to such intensive selection for increased body mass (selection has mainly focused on reproductive traits) and its growth rate is similar to that of the mallard. Therefore, it is a useful baseline for comparison with the broiler (Fig. 1).

The length of the tibiotarsus scales differently in both species (Table 1); the leg bones of all duck lines undergo a similar rapid early development which is in contrast to that of the chicken lines. The duck’s tibiotarsal and femoral growth begins to plateau at 5 weeks of age whereas the chicken’s leg bones continue to grow. A similar pattern of growth is seen in leg muscle mass (Fig. 2b). In other words, leg growth displays positive allometry in chickens and negative allometry in ducks (Table 1). These findings are consistent with a previous study of mallard ontogeny, which demonstrated that leg development plateaus to a level close to that of the adult at 4 weeks post-hatch whereas wing development does not really begin until 3 weeks post-hatch [29]. These alternate strategies of leg development may be due to differences in the behavioural ecology of the birds’ wild ancestors. Predation on chicks represents an intensive selective pressure. The standard predator escape mechanism for ducklings is to run to water and swim away from the bank [30], whereas for jungle fowl chicks, the predator escape mechanism involves periods of immobility and short bursts of flight [31], neither of which require intensive or prolonged use of the legs. Therefore, there may have been a higher selective pressure for well-developed legs early in life in the duck ancestor, which would explain the patterns of hindlimb growth observed in both the mallard and the Pekin lines.

### Tibiotarsal morphology

**Fig. 6:**
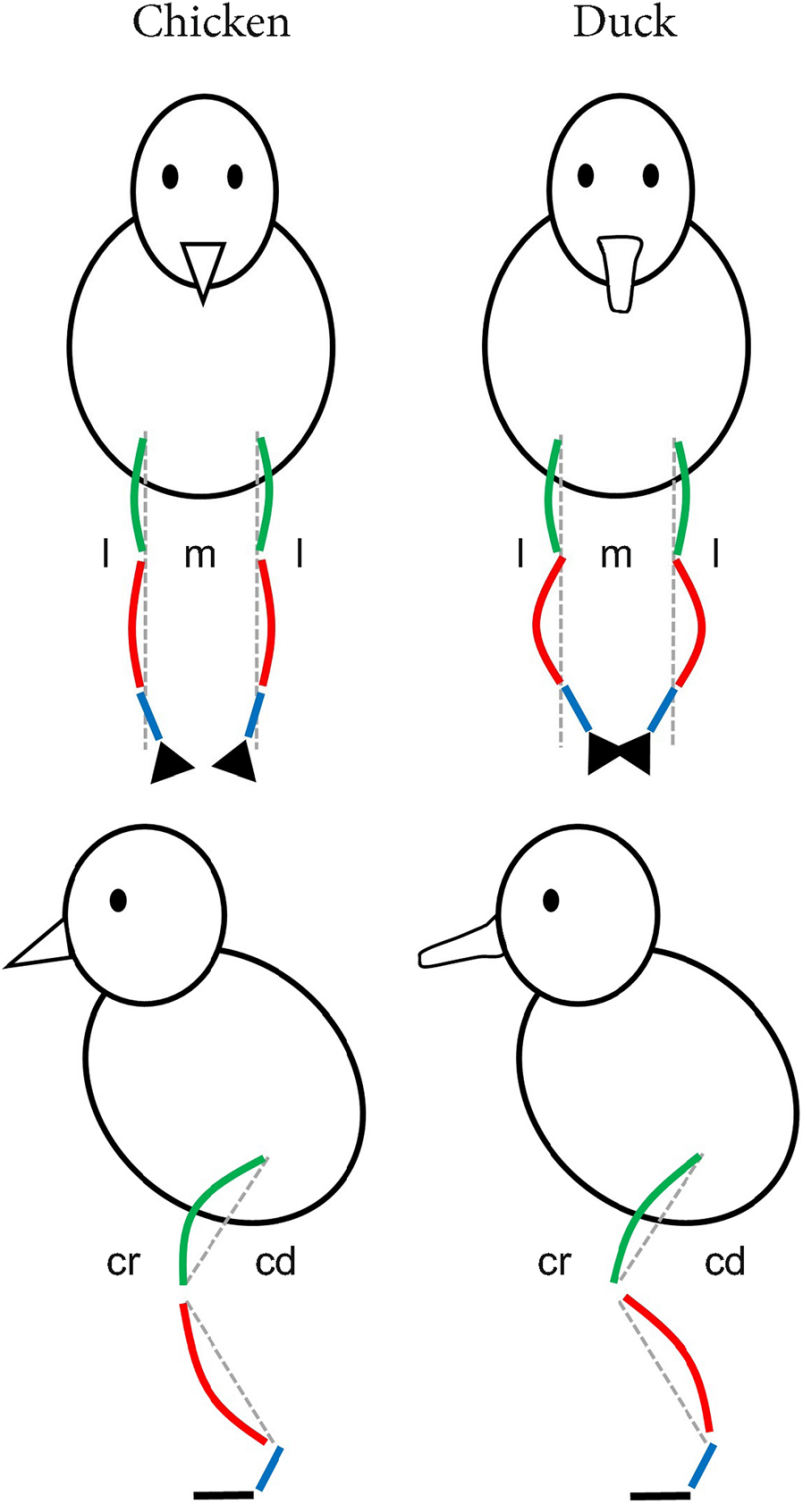
Curvature of the femur (*green*) and tibiotarsus (*red*) in the chicken and duck, shown in both *frontal* and *lateral views*. The tarsometatarsus (*blue*) is represented by a *straight line* since curvatures were not recorded on this bone. Curvatures are exaggerated for clarity. Note the increased lateral curvature of the duck tibiotarsi (and subsequent foot placement) in the medio-lateral plane and the differing directions of tibiotarsal curvature in the cranio-caudal plane. *l* lateral, *m* medial, *cr* cranial, *cd* caudal

Curvature of the tibiotarsus in the cranio-caudal plane differs between both species (Fig. 3c). In chickens, the birds selected for rapid growth rate are more cranially curved than their slow-growing conspecifics but this is not the case in ducks, for which all lines display a similar curvature. Whereas the increased curvature observed in the broiler chicken may be a side-effect of the rapid growth rate, it is not clear why a similar effect does not occur in the Pekin duck lines. The divergence of bone angulation in different directions from 180° (a straight bone) as observed in each species may represent an adaptation to specialised leg use in the ancestor such as paddling in ducks or cursorial or perching behaviour in chickens (Fig. 6).

**Fig. 7:**
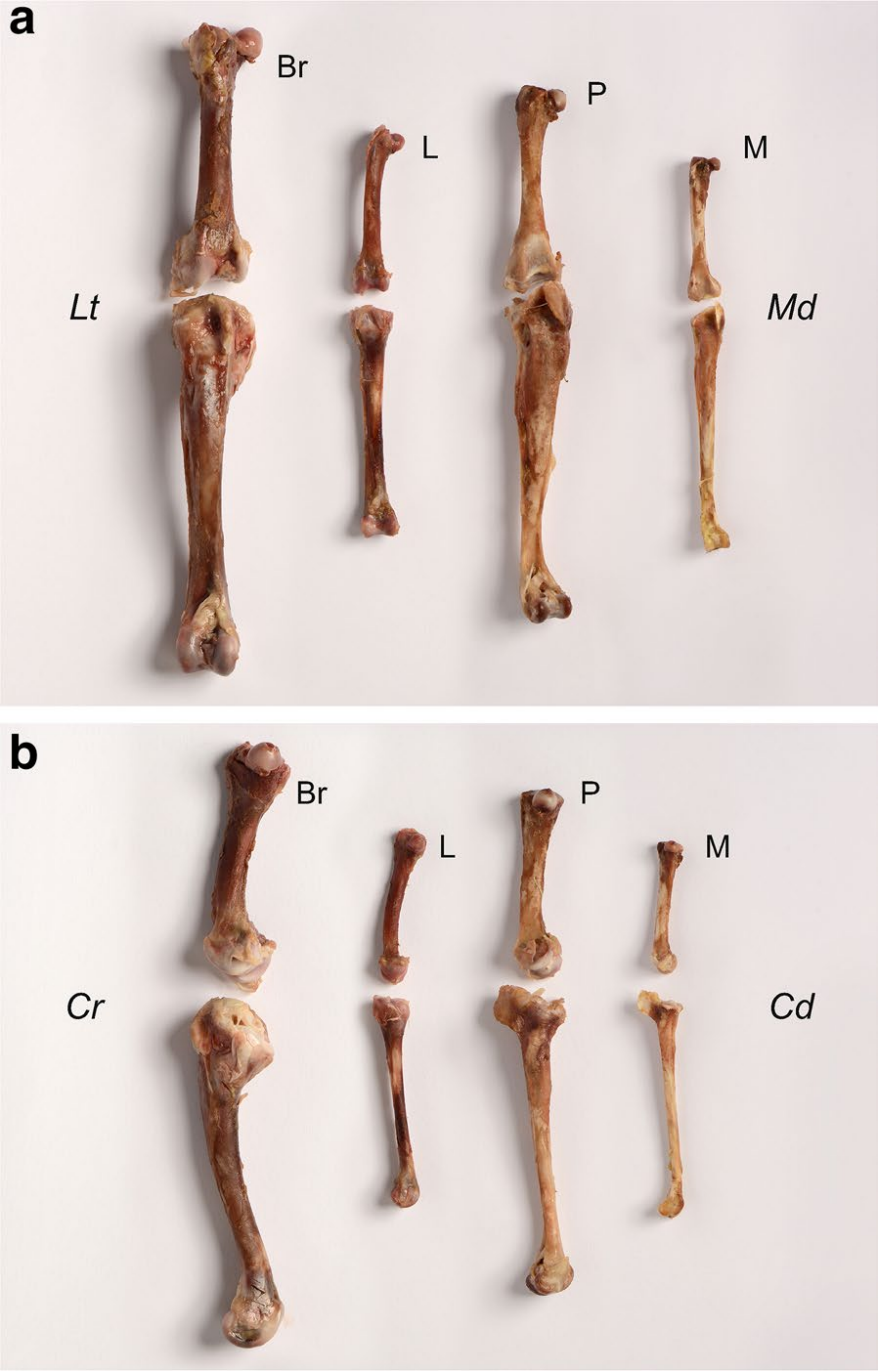
Curvature of the femur (*above*) and tibiotarsus (*below*) in the broiler chicken (*Br*), layer chicken (*L*), Pekin commercial hybrid (*P*) and the mallard (*M*), in both *frontal* (**a**) and *lateral* (**b**) views. These bones were taken from birds aged 7 weeks. Note the lateral curvature of both the Pekin and mallard tibiotarsi (**a**) and the extreme cranial curvature of the broiler tibiotarsus (**b**)

In the medio-lateral plane of the tibiotarsus, ducks selected for rapid growth rate experience more lateral curvature than their ‘unselected’ conspecifics, the mallards (Fig. 3d). This may be a side-effect of rapid growth (although it was not observed in broiler chickens), or it may have developed as an adaptation to loading through the limb. Lateral bending of the tibiotarsus would increase the angle that the tarsometatarsus makes with the sagittal plane of the body, thereby moving the foot to a more medial position which would place the foot under the centre of mass during stance time and thus, increase stability. Divergence for this trait is also observed between species, with the duck lines displaying more curved tibiotarsi (Fig. 7). This suggests that greater lateral curvature may be beneficial to the duck but not to the chicken. Simplistically, the varus deviation of tibiotarsi in ducks would permit the feet to be positioned in a more medially aligned position when they paddle, given that swimming birds typically paddle with their tibiotarsi positioned in a more abducted position than when they walk [32]. The angles of the distal tibiotarsal (intertarsal) joint plane have been reported to differ between the ringed teal (a semi-aquatic species) and the quail (a cursorial species), which supports our findings on tibiotarsal bending in the medio-lateral plane [33]. A lateral curvature of the distal tibiotarsus (Fig. 6) would lead to a change in the angle of the intertarsal joint plane and, thus, affect the position of the tarsometatarsus and move the foot to a more medial position. In guinea fowl, during walking the tibiotarsus and tarsometatarsus are adducted so that the foot remains underneath the centre of mass during stance [34]. Gatesy [35] suggested that the tibiotarsus moves laterally (abducts) to bring the protracting foot clear of the stance limb during its swing phase before adducting again for ground contact. The lateral curvature observed in the duck may be a swimming adaptation which hinders this process during walking. Previous work has demonstrated that unperturbed mallards swim at speeds which minimise the energetic cost of transport [36]. Mallard ducklings will swim in formation which reduces their energy expenditure and it has been suggested that while this is partly due to the drag wake of the leading ducklings, energy may also be ‘recycled’ from vortices shed during the power phase of the lead ducking’s paddling stroke [37]. The lateral curvature of the duck tibiotarsi may assist in harvesting the energy from these shedding vortices to reduce the energetic cost of swimming, but clearly more detailed investigation is required to confirm this. Bone curvature was not analysed allometrically since this trait does not change with increasing body size (Fig. 3).

Rotation of the leg bones can have a major effect on the orientation of some more distal elements of the limb and, thus, greatly influence gait dynamics. The tibiotarsi of both species show a similar degree of torsion at 3 weeks of age (Fig. 4b). However, as ducks grow older, their tibiotarsi twist with the distal end rotating internally. Allometric analysis (Table 1) shows that the scaling exponent of the tibiotarsal torsion in duck lines deviates further from the expected value of zero than that in chickens (here, a negative scaling exponent indicates an increase in internal rotation, rather than negative allometry). The R^2^ values of the regressions for these traits were very close to zero in both chicken lines, which suggests that bone torsion does not scale to body mass in this species. This rotation occurs earlier in the Pekin commercial hybrid than in the male and female Pekin lines and the mallard, a difference which is probably associated with early maturity (Fig. 4b). It is not known why the tibiotarsi of the duck lines rotate as the ducks develop, but one effect of this rotation would be to position the foot more medially during stance and during swimming. In effect, tibiotarsal rotation in this case is complementing the lateral curvature discussed previously. However, if this was a swimming adaptation, one would expect this rotation to occur earlier when a high selection pressure on swimming ability exists in ducklings due to predation (if the same morphological constraints on swimming ability exist across ages). Previous studies have reported a link between tibiotarsal torsion and rapid growth rate [27] but this explanation is contradicted by the observation that duck lines with rapid and slow growths display similar ranges of tibiotarsal rotation. Finally, it is also worth noting that these measures of torsion are calculated using bone landmarks; changes in the relative size/position of these landmarks throughout development may affect the amount of torsion recorded and also affect comparisons between lines.

### Tibiotarsal bone quality

Selection for production traits was expected to affect aspects of the leg which would normally be subjected to a strong level of natural selection. The stiffness of the tibiotarsi in all lines scaled with isometry (geometrically similar to body mass) with the exception of the layer chicken, which scaled with very positive allometry (Table 1). This may be a strategy to counteract the comparatively small radius of the layer bones. The radius of a bone exponentially affects its strength, so relatively narrow bones (such as those of the layer) will be exponentially weaker. Surprisingly, the stiffness of the mallard bones does not scale with a similarly positive allometry. In chickens, the cortical area of the tibiotarsus increased at a faster rate than the rest of the body; in ducks the tibiotarsal cortical area displayed negative allometry (with the exception of the Pekin hybrid, which grew isometrically). This is another indication that legs reach adult size and slow down their growth earlier in the duck lines than in the chicken lines. The tibiotarsi of the broiler, layer, Pekin hybrid and mallard became relatively stronger as the bird grew; the maximum load tolerated by the tibiotarsi before breaking scaled positively in these lines (the male and female Pekin breeding lines scaled isometrically). The range of bending stresses measured in the tibiotarsi during breaking were similar for all lines, which suggests that the birds, regardless of their size, adapt their bone morphology in a similar way to suit the forces subjected on them (Fig. 5b). However, by 7 weeks of age, the tibiotarsi of the duck lines tolerated more stress than those of the chicken lines, which indicates that either the selection pressure on chicken lines for production traits has occurred at the expense of the mechanical properties of their bones, or that the composition of bone rather than its gross morphology allowed the bones of the duck lines to tolerate relatively high forces. The mallard tibiotarsi tolerated slightly more bending stress than those of the selected duck lines, which would support this theory. Also, at this developmental stage, the chicken tibiotarsi are still growing, which may explain why they are not as mechanically robust as those of the duck. The growth of the duck tibiotarsi is considerably slower at this stage, which allows them more opportunity to remodel to handle the loads imposed on them. Previous studies on broiler chickens suggested that lines selected for rapid growth, while having tibiotarsi of the correct dimensions for supporting greater loads, have greater porosity and lower levels of cortical bone mineralisation than slower growing lines [22, 23].

Differences in bone mineral content did not explain the mechanical changes; all lines showed an increase in mineralisation of the mid-diaphyseal tibiotarsus until 5 weeks of age, and thereafter the rate of mineralisation stabilised (Fig. 5c). The differential mechanical properties of avian bone were consistent with the histological measures of porosity. The duck tibiotarsi became less porous (and thus stronger) as the birds aged, allowing them to tolerate greater bending stresses, whereas the chicken lines maintained the same levels of tibiotarsal porosity throughout the same growth period (Fig. 5d). Neither bending stress nor porosity scaled to body mass. Bending stress is a metric which has already been corrected for body mass in its calculation, and thus it is expected to show no relationship with body mass. It is likely that porosity is mainly influenced by genotype and environmental factors (such as feed) rather than by the size of the bird (although loading forces acting on the bone due to body mass will affect porosity through bone remodelling).

These findings on the divergence of pelvic limb morphology within two species of poultry provide useful information, which can be used to lay the foundations for further investigations on the link between anatomy and gait in poultry.

## Conclusions

It is clear that artificial selection for increased growth rate has resulted in diverging hindlimb architectures within species that have been domesticated. Natural selection that acted on these species prior to domestication, has also affected leg morphology. Since the terrestrial lifestyle of the domestic duck differs from that of its semi-aquatic ancestors, it is possible that some hindlimb adaptations for aquatic locomotion, such as lateral curvature of the tibiotarsus, may be a hindrance to effective terrestrial locomotion in the commercial Pekin duck. Indeed, it is interesting that the Pekin duck can ambulate with relative ease compared to the broiler chicken, which reaches a similar size in the same growth period. Future investigations on the differences in leg morphology between strictly cursorial species such as the chicken and swimmers such as ducks may shed some light on these adaptations and their possible effects on gait.

## Additional files

10.1186/s12711-015-0166-9 A more detailed description of the methods presented in the main text.
10.1186/s12711-015-0166-9 Tables of least squares means for each trait measured on all lines at each age.

## References

[1] FAOSTAT (database on the Internet). FAO. 2014. http://faostat.fao.org/default.aspx. Accessed 20 May 2015.

[2] Corr SA, Gentle MJ, McCorquodale CC, Bennett D (2003). The effect of morphology on the musculoskeletal system of the modern broiler. Anim Welf.

[3] Schmidt CJ, Persia ME, Feierstein E, Kingham B, Saylor WW (2009). Comparison of a modern broiler line and a heritage line unselected since the 1950s. Poult Sci.

[4] Knowles TG, Kestin SC, Haslam SM, Brown SN, Green LE, Butterworth A (2008). Leg disorders in broiler chickens: prevalence, risk factors and prevention. PLoS One.

[5] Paxton H, Daley MA, Corr SA, Hutchinson JR (2013). The gait dynamics of the modern broiler chicken: a cautionary tale of selective breeding. J Exp Biol.

[6] Kestin SC, Knowles TG, Tinch AE, Gregory NG (1992). Prevalence of leg weakness in broiler chickens and its relationship with genotype. Vet Rec.

[7] Sanotra GS, Berg C, Lund JD (2003). A comparison between leg problems in Danish and Swedish broiler production. Anim Welf.

[8] Sanotra GS, Lund JD, Ersboll AK, Petersen JS, Vestergaard KS (2001). Monitoring leg problems in broilers: a survey of commercial broiler production in Denmark. Worlds Poult Sci J.

[9] EFSA Panel on Animal Health and Welfare (2010). Scientific opinion on the influence of genetic parameters on the welfare and the resistance to stress of commercial broilers. EFSA J..

[10] Yogaratnam V (1995). Analysis of the causes of high rates of carcass rejection at a poultry processing plant. Vet Rec..

[11] McGeown D, Danbury TC, Waterman-Pearson AE, Kestin SC (1999). Effect of carprofen on lameness in broiler chickens. Vet Rec.

[12] Danbury TC, Weeks CA, Chambers JP, Waterman-Pearson AE, Kestin SC (2000). Self-selection of the analgesic drug carprofen by lame broiler chickens. Vet Rec..

[13] Caplen G, Colborne GR, Hothersall B, Nicol CJ, Waterman-Pearson AE, Weeks CA (2013). Lame broiler chickens respond to non-steroidal anti-inflammatory drugs with objective changes in gait function: a controlled clinical trial. Vet J..

[14] Vestergaard KS, Sanotra GS (1999). Relationships between leg disorders and changes in the behaviour of broiler chickens. Vet Rec..

[15] Weeks CA, Danbury TD, Davies HC, Hunt P, Kestin SC (2000). The behaviour of broiler chickens and its modification by lameness. Appl Anim Behav Sci..

[16] Kapell DNRG, Hill WG, Neeteson AM, McAdam J, Koerhuis ANM, Avendano S (2012). Twenty-five years of selection for improved leg health in purebred broiler lines and underlying genetic parameters. Poult Sci.

[17] Jones TA, Dawkins MS (2010). Environment and management factors affecting Pekin duck production and welfare on commercial farms in the UK. Br Poult Sci.

[18] Rodenburg TB, Bracke MBM, Berk J, Cooper J, Faure JM, Guémené D (2005). Welfare of ducks in European duck husbandry systems. Worlds Poult Sci J..

[19] Corr SA, Gentle MJ, McCorquodale CC, Bennett D (2003). The effect of morphology on walking ability in the modern broiler: a gait analysis study. Anim Welf.

[20] Paxton H, Tickle PG, Rankin JW, Codd JR, Hutchinson JR (2014). Anatomical and biomechanical traits of broiler chickens across ontogeny. Part II. Body segment inertial properties and muscle architecture of the pelvic limb. Peer J.

[21] Thorp BH (1994). Skeletal disorders in the fowl—a review. Avian Pathol..

[22] Williams B, Solomon S, Waddington D, Thorp B, Farquharson C (2000). Skeletal development in the meat-type chicken. Br Poult Sci.

[23] Williams B, Waddington D, Murray DH, Farquharson C (2004). Bone strength during growth: influence of growth rate on cortical porosity and mineralization. Calcif Tissue Int.

[24] Van Wyhe RC, Applegate TJ, Lilburn MS, Karcher DM (2012). A comparison of long bone development in historical and contemporary ducks. Poult Sci.

[25] Allen V, Elsey RM, Jones N, Wright J, Hutchinson JR (2010). Functional specialization and ontogenetic scaling of limb anatomy in *Alligator mississippiensis*. J Anat.

[26] Paxton H, Anthony NB, Corr SA, Hutchinson JR (2010). The effects of selective breeding on the architectural properties of the pelvic limb in broiler chickens: a comparative study across modern and ancestral populations. J Anat.

[27] Bradshaw RH, Kirkden RD, Broom DM (2002). A review of the aetiology and pathology of leg weakness in broilers in relation to welfare. Avian Poult Biol Rev..

[28] Leterrier C, Nys Y (1992). Composition, cortical structure and mechanical properties of chicken tibiotarsi: effect of growth rate. Br Poult Sci.

[29] Dial TR, Carrier DR (2012). Precocial hindlimbs and altricial forelimbs: partitioning ontogenetic strategies in mallards (*Anas platyrhynchos*). J Exp Biol.

[30] Dial TR, Heers AM, Tobalske BW (2012). Ontogeny of aerodynamics in mallards: comparative performance and developmental implications. J Exp Biol.

[31] Collias NE, Collias EC (1967). A field study of the red jungle fowl in north-central India. Condor.

[32] Provini P, Goupil P, Hugel V, Abourachid A (2012). Walking, paddling, waddling: 3D kinematics Anatidae locomotion (*Callonetta leucophrys*). J Exp Zool A Ecol Genet Physiol..

[33] Provini P, Simonis C, Abourachid A (2013). Functional implications of the intertarsal joint shape in a terrestrial (*Coturnix coturnix*) versus a semi-aquatic bird (*Callonetta leucophrys*). J Zool.

[34] Abourachid A, Hackert R, Herbin M, Libourel PA, Lambert F, Gioanni H (2011). Bird terrestrial locomotion as revealed by 3D kinematics. Zoology (Jena)..

[35] Gatesy SM (1999). Guineafowl hind limb function. I: cineradiographic analysis and speed effects. J Morphol.

[36] Prange HD, Schmidt-Nielsen K (1970). The metabolic cost of swimming in ducks. J Exp Biol.

[37] Fish FE (1995). Kinematics of ducklings swimming in formation: consequences of position. J Exp Zool.

