# Supplementary material for: Differences in hindlimb morphology of ducks and chickens: effects of domestication and selection
Source: Genet Sel Evol. 2015 Nov 17;47:88. doi: 10.1186/s12711-015-0166-9 (PMC4647608; doi:10.1186/s12711-015-0166-9)
Supplement: Supplementary file 1 — 10.1186/s12711-015-0166-9 A more detailed description of the methods presented in the main text. [file 12711_2015_166_MOESM1_ESM.docx]

Supplementary methods

**Bone morphology measurement by computed tomography**

All bones were evaluated with a computed tomography (CT) scan using a ‘Somatom Volume Zoom’ helical 4-slice CT unit (Siemens, Germany). A helical scan of the intact femur and tibiotarsus was performed with the following settings: 120kV, 130 mAs, 1.5s tube rotation time, 1mm slice width, collimator pitch of 0.75, high resolution image reconstruction kernel (proprietary name U90u).

Bone morphology was assessed using a dedicated DICOM viewing software (Osirix, Geneva, Switzerland, version 5.8.5-32bit) on a computer workstation (Apple IMac 27 inch, Apple, USA) with a calibrated monitor. The multiplanar reconstruction application of the software was used for angular and other measurements. Morphological measurements taken for both femur and tibiotarsus included functional length, diameter and cortical cross-sectional area at the mid-diaphysis, curvature in both frontal and sagittal planes and torsion.

Length was measured in the cranio-caudal plane by drawing a straight line between the midpoint of the distal end of the bone to the midpoint at the proximal end of the bone.

The mid-diaphysis was located by halving the length measure above. As the cross section (in the transverse plane) of the bone is approximately elliptical in shape, the lengths of both the major and minor axes were recorded. Area of the whole cross-section of bone at the mid-diaphysis was measured using the ‘area’ tool within the software by highlighting the whole cross-sectional area. Internal area was measured by highlighting the non-cortical area of the cross-section. This was subtracted from the total area to obtain the cortical area of the bone at the mid-diaphysis.

To measure curvature in the cranio-caudal plane, the midpoint of the bone was located by halving the distance between the lateral periosteal surfaces of the mid-diaphysis when viewed in the cranio-caudal plane. Two lines were drawn from this midpoint of the mid-diaphysis – one to the midpoint of the distal end and another to the midpoint of the proximal end. The caudal angle made by these two lines was recorded as the curvature of the bone in the cranio-caudal plane. The same process was used to measure curvature in the medio-lateral plane, with the medial angle measured in this plane.

Bone torsion was measured by creating a separate DICOM file in the transverse plane of the bone, covering its whole length. A slice from the distal end was then overlaid on a slice from the proximal end of the bone. A line was drawn between selected landmarks chosen for each site. The angle between these two lines (one line drawn on the distal slice and one line on the proximal slice) was recorded as the relative torsion of the whole bone. Details of which slices were compared and which landmarks were used to form each line are given in Table S1. An example of how these lines were used to calculate relative torsion is shown in Figure S5.

| **Bone** | **Figure** | **Position along proximal-distal axis** | **Landmarks** |
| --- | --- | --- | --- |
| Proximal femur | S1 | The slice at which the diameter of the femoral head is greatest. | A line joining the centre of the femoral head to the most cranial point of the *trochanter femoris*. |
| Distal femur | S2 | The slice at which both the *condylus lateralis* and *condylus medialis* are at their most pronounced. | A line joining the most caudal points of the *condylus lateralis* and *condylus medialis* |
| Proximal tibiotarsus | S3 | Approaching from the proximal end, the slice at which the first sign of mineralisation appears in the *facies articularis femoralis*. | A line joining the most caudal point of the *crista cnemialis cranialis* with the most caudal point of the *facies articularis lateralis*. |
| Distal tibiotarsus | S4 | The slice at which both the *condylus lateralis* and *condylus medialis* are at their most pronounced. | A line joining the most caudal points of the *condylus lateralis* and *condylus medialis* |

Table S1. Anatomical landmarks used to calculate bone torsion

Details of anatomical terms used given in The Anatomical Atlas of *Gallus* [[1](#_ENREF_1)].


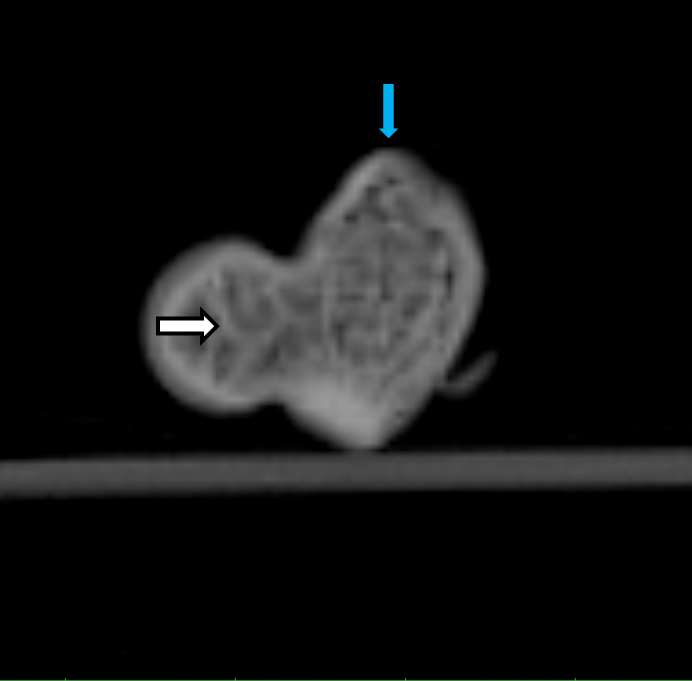


Fig S1. Proximal femur in cross-section, with the centre of the femoral head (white arrow) and *trochanter femoris* (blue arrow) highlighted.


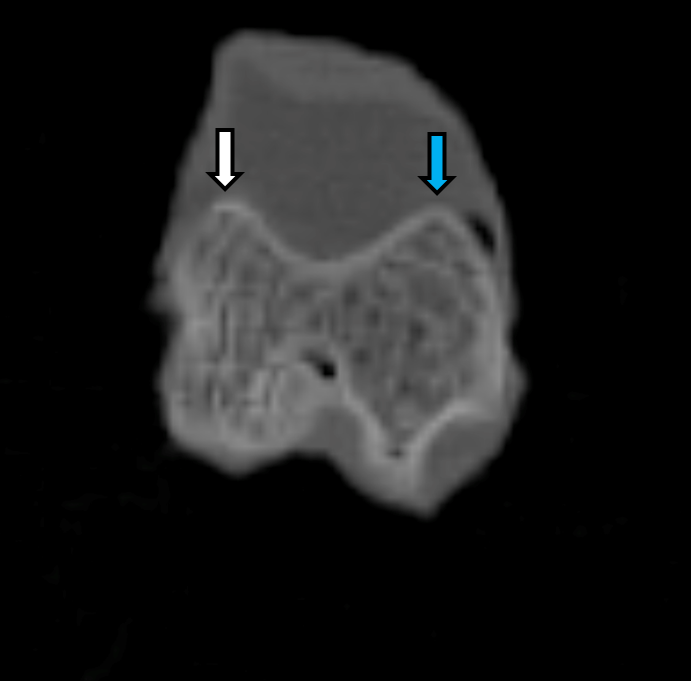


Fig S2. Distal femur in cross-section, with the *condylus lateralis* (white arrow) and *condylus medialis* (blue arrow) highlighted.


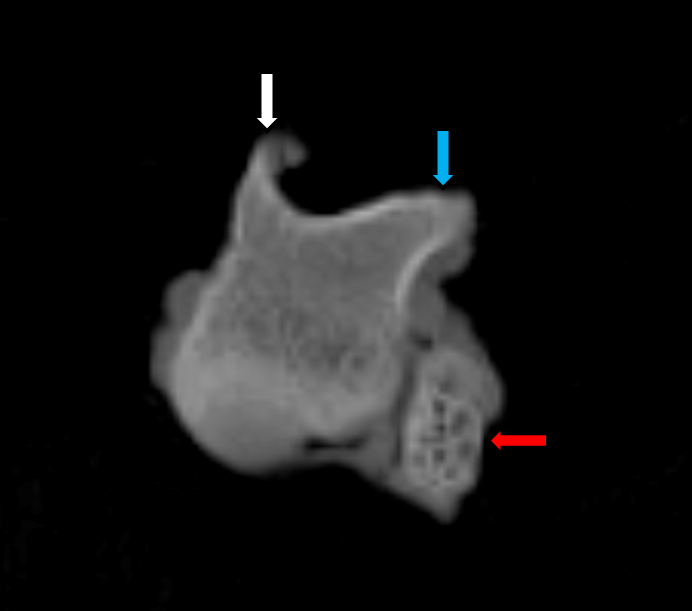


Fig S3. Proximal tibiotarsus in cross-section, with the *crista cnemialis cranialis* (white arrow), the *facies articularis lateralis* (blue arrow) and the mineralised *facies articularis femoralis* (red arrow) highlighted.


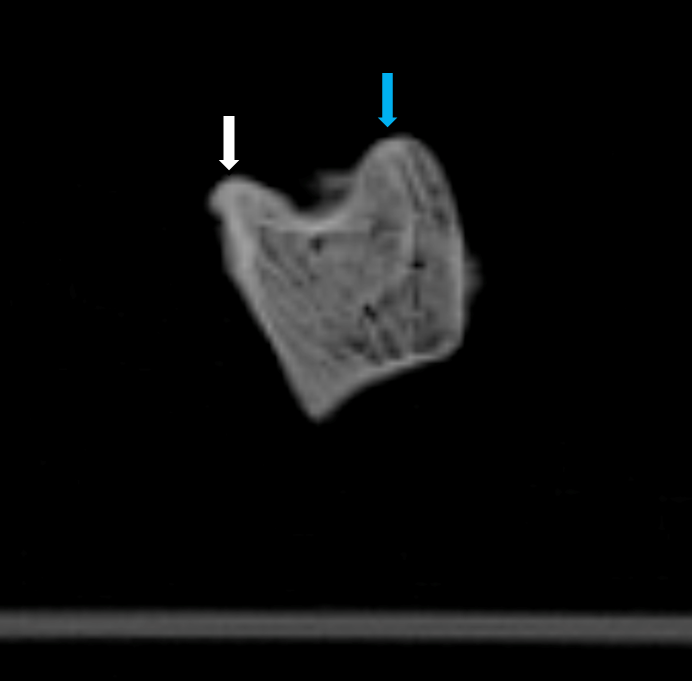


Fig S4. Distal tibiotarsus in cross-section, with the *condylus lateralis* (white arrow) and *condylus medialis* (blue arrow) highlighted.


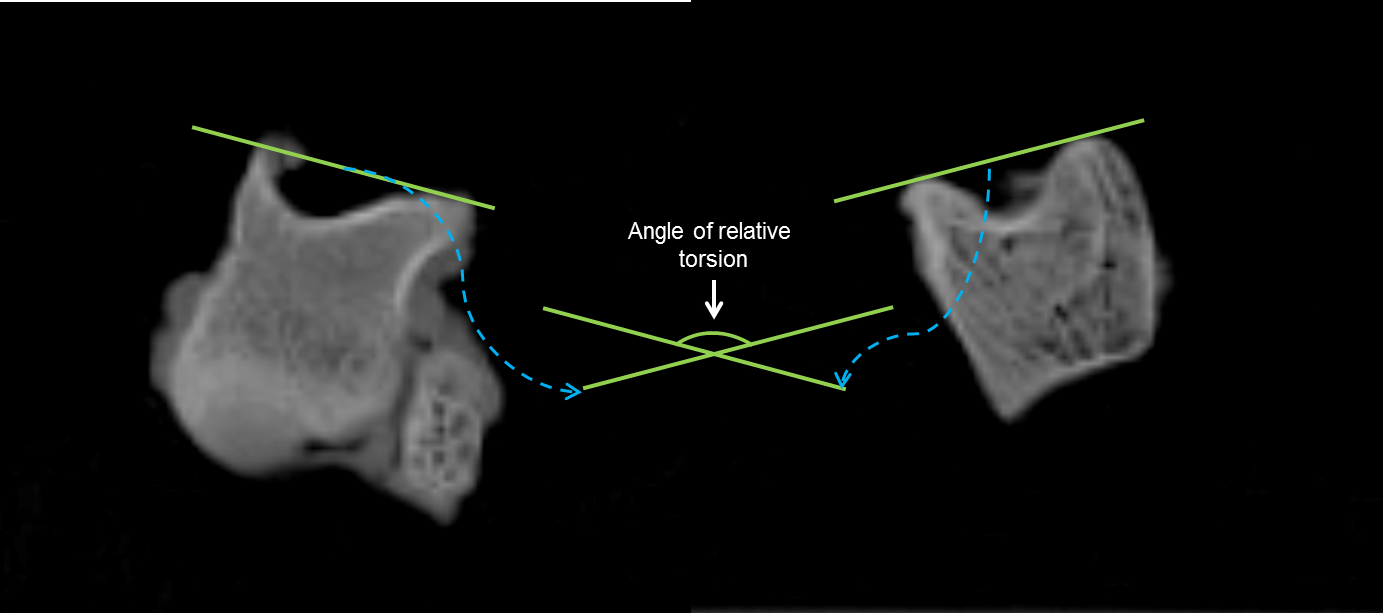


Fig S5. An example of how the angle of relative bone torsion is calculated in the tibiotarsus.

**Bending stress calculations**


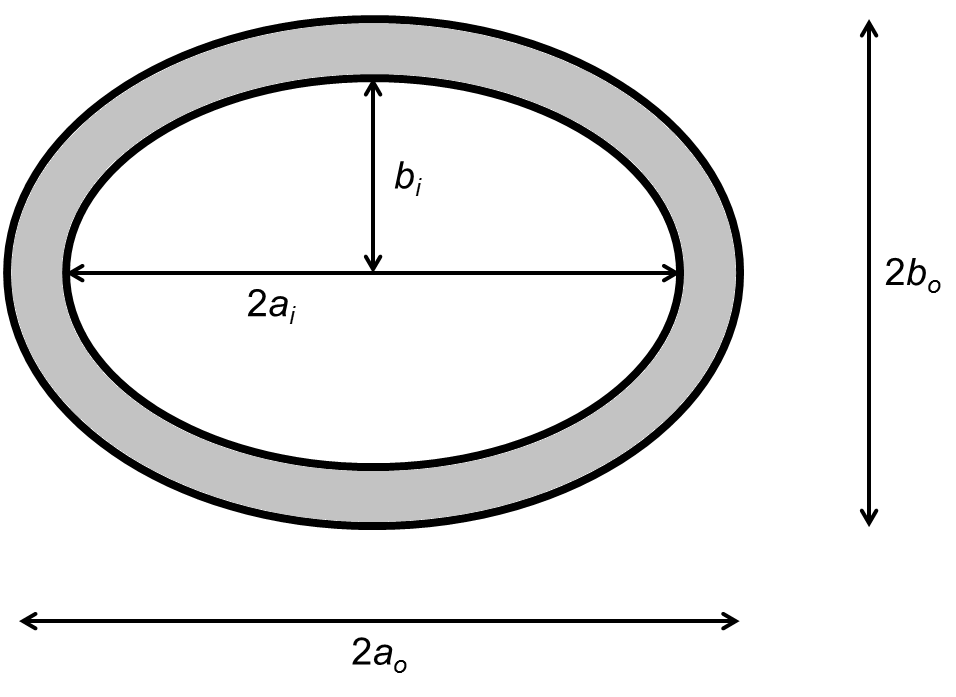


Fig. S6. Schematic of mid-diaphyseal tibiotarsal cross-section showing measurements taken or deduced as below.

The second area moment of inertia (*I*) was calculated by assuming the bone to be a perfect hollow elliptical cylinder of constant wall thickness and outer semi-major axis a_o_, inner semi-major axis a_i_, outer semi-minor axis b_o_ and inner semi-minor axis b_i_ as:

$I= \frac{\pi}{4}\left( a_{o}{b_{o}}^{3}- a_{i}{b_{i}}^{3} \right)$ … *a* > *b* [[2](#_ENREF_2)]

This formula to calculate the second area moment of inertia (*I*) requires the assumption of a constant wall thickness. Therefore, rather than using measurements of *a_i_* and *b_i_* from CT analysis, it is assumed that $a_{o}: b_{o}= a_{i}: b_{i}$

Assume $a_{o}: b_{o}=x$

So if $a_{o}=xb_{o}$then $a_{i}=xb_{i}$

$${Area}_{i}= \pi a_{i}b_{i}$$

$$A_{i}= \pi{xb}_{i}b_{i}$$

$$\frac{A_{i}}{\pi x}= {b_{i}}^{2}$$

$$b_{i}= \sqrt{\frac{A_{i}}{\pi x}}$$

$$a_{i}=xb_{i}$$

Both *a_o_* and *b_o_* and taken from CT measurements.

The second area moment of inertia (*I*) is then used to calculate the bending stress (*B*) experienced by the bone, using the formula given in the main text.

**Histology protocol for porosity measurement**

Staining and mounting was carried out by the Veterinary Services department at the Royal (Dick) School of Veterinary Studies, UK.

To assess the porosity of each bone, 1 cm samples of the mid-diaphysis of the left tibiotarsus from each bird were cut using a bench based bone saw. The tissues were placed into 10% buffered formalin for about a week followed by decalcification in Decal I solution from Leica until soft enough to cut through easily. They were checked every 3 days until soft. The tissue was then washed in running water to remove the decal fluid for 1 hour, then processed over 2 days through graded ethanol xylenes and fixed into wax. Sections were cooled on ice and cut at 3 microns. Sections were picked up onto electrostatically charged microscope slides. The sections were dried overnight at 37°C then heated to 60°C for 30 minutes before staining with haematoxylin and eosin.

Porosity was measured on a Leica DMRB fluorescent microscope using a x5 objective lens. Photographs were taken of north, south, east and west sections of cortical bone mounted on slides. Porosity was then calculated for each photograph by converting the image to a greyscale 32bit image and counting the percentage of pixels in each image which were black (the percentage porosity) using the ‘analyse particles’ function within ImageJ, a public domain image analysis software (NIH, USA, version 1.6.0_20, 64-bit). Porosity results of the north, south, east and west sections of each bird were averaged to obtain an overall cortical porosity for each bird.

**Mineral content analysis protocol**

Bone mineral analysis was carried out by DM Scientific (Thirsk, UK).

To measure the mineral content of each bone, 2 cm samples of the mid-diaphysis of the right tibiotarsus were cut using a bench based bone saw. Bone samples were stored at -20°C and defrosted prior to starting analysis.

A 1cm portion was taken and weighed on a 4 place balance. This 1cm portion was dried in an oven at 103°C, the bone was then cooled and weighed to calculate the dry matter. To defat, the sample was wrapped in a filter paper and placed in a soxhlet extraction apparatus and extracted for four hours under reflux with petroleum ether. The sample was then air dried overnight and placed in an oven at 103°C for four hours to remove any residual solvent.

Ash content was calculated by placing the sample into a pre-weighed ash crucible and heating overnight in a furnace at 550°C. Once removed and cooled the sample and crucible was weighed to obtain the ash result.

The resulting ash sample was then used for the calcium and phosphorus analysis.

Sample was placed in a beaker and heated with 50% hydrochloric acid; once cooled the solution was placed in a 250ml volumetric flask and diluted with deionised water.

To determine calcium concentration, the sample solution was diluted in a strontium chloride solution and determined on an atomic absorption spectrometer using a standard calibration curve.

To calculate phosphorus content the sample solution was diluted in an ammonium molybdovanadate solution and determined on a visible spectrometer at 430nm using a standard calibration curve.

References

1. Yasuda M. The Anatomical Atlas of Gallus. Tokyo: Unviversity of Tokyo Press; 2002.

2. Utz JC, Nelson S, O'Toole BJ, van Breukelen F. Bone strength is maintained after 8 months of inactivity in hibernating golden-mantled ground squirrels, Spermophilus lateralis. J Exp Biol. 2009;212(17):2746-52. doi:10.1242/jeb.032854.
