# Supplementary material for: Differences in hindlimb morphology of ducks and chickens: effects of domestication and selection
Source: Genet Sel Evol. 2015 Nov 17;47:88. doi: 10.1186/s12711-015-0166-9 (PMC4647608; doi:10.1186/s12711-015-0166-9)
Supplement: Supplementary file 2 — 10.1186/s12711-015-0166-9 Tables of least squares means for each trait measured on all lines at each age. [file 12711_2015_166_MOESM2_ESM.docx]

Supplementary Results

| **Line** | **Length (cm)** | | | **Cranio-caudal curvature (°)** | | | **Medio-lateral curvature (°)** | | | **Torsion (°)** | | | **Cortical Area (cm^2^)** | | |
| --- | --- | --- | --- | --- | --- | --- | --- | --- | --- | --- | --- | --- | --- | --- | --- |
| *Experiment 1* | *3 wks* | *5 wks* | *7 wks* | *3 wks* | *5 wks* | *7 wks* | *3 wks* | *5 wks* | *7 wks* | *3 wks* | *5 wks* | *7 wks* | *3 wks* | *5 wks* | *7 wks* |
| **Broiler chicken** | 7.59 | 10.24 | 11.72 | 175.5 | 174.6 | 174.4 | 176.7 | 175.5 | 175.9 | 174.2 | 167.8 | 170.1 | 0.18 | 0.35 | 0.37 |
| **Layer chicken** | 5.49 | 6.82 | 8.69 | 179.1 | 178.7 | 179.5 | 176.9 | 176.8 | 177.2 | 173.6 | 174.2 | 174.6 | 0.06 | 0.10 | 0.13 |
| **Pekin hybrid** | 10.25 | 11.35 | 11.57 | 183.2 | 183.0 | 181.4 | 172.2 | 172.7 | 172.1 | 166.3 | 153.5 | 153.9 | 0.15 | 0.21 | 0.23 |
| SED | 0.16  P<0.001 df=4 | | | 0.74  P=0.151 df=4 | | | 0.66  P=0.285 df=4 | | | 3.08  P=0.005 df=4 | | | 0.02  P<0.001 df=4 | | |
| *Experiment 2* | *3 wks* | *5 wks* | *7wks* | *3 wks* | *5 wks* | *7wks* | *3 wks* | *5 wks* | *7wks* | *3 wks* | *5 wks* | *7wks* | *3 wks* | *5 wks* | *7 wks* |
| **Pekin male line** | 9.35 | 10.79 | 10.93 | 181.3 | 181.6 | 180.6 | 172.7 | 171.7 | 172.5 | 168.2 | 167.1 | 151.3 | 0.14 | 0.18 | 0.20 |
| **Pekin female line** | 9.22 | 10.81 | 10.84 | 183.0 | 182.5 | 182.0 | 172.2 | 172.3 | 172.5 | 175.0 | 169.6 | 157.5 | 0.12 | 0.15 | 0.17 |
| **Mallard** | 7.54 | 8.43 | 8.73 | 182.4 | 182.9 | 181.9 | 174.6 | 174.3 | 174.4 | 171.9 | 165.4 | 154.2 | 0.08 | 0.09 | 0.10 |
| SED | 0.11  P=0.001 df=4 | | | 0.71  P=0.887 df=4 | | | 0.65  P=0.756 df=4 | | | 2.86  P=0.712 df=4 | | | 0.02  P=0.001 df=4 | | |

Table S2: Tibiotarsal morphology (Least squares means and standard errors of difference) in all lines at each age.

| **Line** | **Stiffness (N/mm)** | | | **Maximum load (N)** | | | **Bending stress (N/cm^2^)** | | | **BMD (%)** | | | **Porosity (%)** | | |
| --- | --- | --- | --- | --- | --- | --- | --- | --- | --- | --- | --- | --- | --- | --- | --- |
| *Experiment 1* | *3 wks* | *5 wks* | *7wks* | *3 wks* | *5 wks* | *7wks* | *3 wks* | *5 wks* | *7wks* | *3 wks* | *5 wks* | *7wks* | *3 wks* | *5 wks* | *7wks* |
| **Broiler chicken** | 224 | 349 | 415 | 284 | 483 | 627 | 951 | 652 | 664 | 38.0 | 47.0 | 44.1 | 25.8 | 22.2 | 25.6 |
| **Layer chicken** | 53 | 130 | 189 | 60 | 121 | 177 | 821 | 717 | 615 | 27.6 | 38.9 | 44.3 | 20.8 | 19.6 | 20.7 |
| **Pekin hybrid** | 183 | 241 | 336 | 193 | 290 | 435 | 614 | 684 | 926 | 37.2 | 39.2 | 41.6 | 22.7 | 19.7 | 16.1 |
| SED | 14.6  P=0.002 df=4 | | | 19.9  P<0.001 df=4 | | | 75.4  P<0.001 df=4 | | | 1.4  P<0.001 df=3 | | | 2.1  P=0.067 df=4 | | |
| *Experiment 2* | *3 wks* | *5 wks* | *7wks* | *3 wks* | *5 wks* | *7wks* | *3 wks* | *5 wks* | *7wks* | *3 wks* | *5 wks* | *7wks* | *3 wks* | *5 wks* | *7wks* |
| **Pekin male line** | 148 | 257 | 333 | 201 | 314 | 410 | 721 | 883 | 781 | 28.8 | 45.0 | 46.5 | 29.0 | 22.9 | 19.2 |
| **Pekin female line** | 158 | 248 | 360 | 171 | 274 | 382 | 645 | 660 | 867 | 32.9 | 44.5 | 45.9 | 28.6 | 19.0 | 15.1 |
| **Mallard** | 168 | 207 | 248 | 108 | 173 | 206 | 827 | 1020 | 1103 | 36.5 | 43.1 | 38.1 | 17.9 | 14.5 | 13.1 |
| SED | 16.2  P<0.001 df=4 | | | 12.1  P<0.001 df=4 | | | 80.1  P=0.019 df=3 | | | 1.4  P<0.001 df=4 | | | 1.7  P=0.012 df=4 | | |

Table S3: Tibiotarsal bone quality (Least squares means and standard errors of difference) in all lines at each age.

| **Line** | **Length (cm)** | | | **Cranio-caudal curvature (°)** | | | **Medio-lateral curvature (°)** | | | **Torsion (°)** | | | **Cortical Area (cm^2^)** | | |
| --- | --- | --- | --- | --- | --- | --- | --- | --- | --- | --- | --- | --- | --- | --- | --- |
| *Experiment 1* | *3 wks* | *5 wks* | *7 wks* | *3 wks* | *5 wks* | *7 wks* | *3 wks* | *5 wks* | *7 wks* | *3 wks* | *5 wks* | *7 wks* | *3 wks* | *5 wks* | *7 wks* |
| **Broiler chicken** | 5.70 | 7.21 | 8.17 | 166.6 | 168.5 | 167.2 | 171.3 | 174.8 | 174.8 | 126.2 | 125.9 | 127.7 | 0.23 | 0.40 | 0.42 |
| **Layer chicken** | 4.01 | 5.01 | 6.21 | 167.6 | 167.2 | 166.9 | 172.0 | 173.4 | 173.8 | 134.9 | 135.7 | 133.6 | 0.07 | 0.12 | 0.16 |
| **Pekin hybrid** | 6.27 | 7.18 | 7.38 | 170.5 | 169.3 | 169.4 | 173.3 | 173.7 | 174.2 | 138.2 | 128.9 | 123.9 | 0.19 | 0.24 | 0.22 |
| SED | 0.10  P<0.001 df=4 | | | 1.06  P=0.428 df=4 | | | 0.80  P=0.094 df=4 | | | 1.86  P<0.001 df=4 | | | 0.02  P<0.001 df=4 | | |
| *Experiment 2* | *3 wks* | *5 wks* | *7wks* | *3 wks* | *5 wks* | *7wks* | *3 wks* | *5 wks* | *7wks* | *3 wks* | *5 wks* | *7wks* | *3 wks* | *5 wks* | *7wks* |
| **Pekin male line** | 5.65 | 6.89 | 7.14 | 171.9 | 173.7 | 174.9 | 170.8 | 173.6 | 174.8 | 137.9 | 130.3 | 124.4 | 0.22 | 0.22 | 0.22 |
| **Pekin female line** | 5.54 | 6.81 | 6.76 | 171.5 | 171.1 | 170.7 | 174.4 | 175.7 | 175.5 | 136.2 | 130.4 | 125.5 | 0.17 | 0.17 | 0.18 |
| **Mallard** | 4.41 | 5.03 | 5.24 | 169.9 | 172.5 | 171.4 | 174.6 | 174.3 | 174.5 | 139.8 | 135.8 | 133.2 | 0.09 | 0.10 | 0.10 |
| SED | 0.07  P<0.001 df=4 | | | 1.07  P=0.1 df=4 | | | 0.71  P<0.001 df=4 | | | 1.9  P=0.084 df=4 | | | 0.01  P=0.589 df=4 | | |

Table S4: Femoral morphology (Least squares means and standard errors of difference) in all lines at each age

Fig. S7: Changes in the length of the left femur with age from Experiment 1 (broiler chicken, layer chicken, Pekin duck commercial hybrid) and Experiment 2 (male line Pekin, female line Pekin and mallard). The upper and lower boxplot whiskers extend to within 1.5 times above and below the interquartile range respectively.

Fig. S8: Molar ratio of Ca:P in mid-diaphyseal cortical bone of the tibiotarsus in each line at each age.
